# Catheter Ablation for Ventricular Tachycardias: Current Status and Future Perspectives

**DOI:** 10.3390/jcm13226805

**Published:** 2024-11-12

**Authors:** Naoya Kataoka, Teruhiko Imamura

**Affiliations:** Second Department of Internal Medicine, University of Toyama, 2630 Sugitani, Toyama 930-0194, Japan; nkataoka@med.u-toyama.ac.jp

**Keywords:** scar-related ventricular tachycardia, conduction delay, repolarization heterogeneity, functional substrate mapping

## Abstract

Catheter ablation for ventricular tachycardia (VT) in patients with systolic heart failure remains a critical yet challenging area of non-pharmacological therapy. Despite positive outcomes in atrial fibrillation, evidence for the efficacy of VT ablation in reducing cardiac mortality is inconclusive due to the absence of standardized ablation strategies. The primary challenges include difficulties in identifying suitable ablation targets and their deep locations within myocardial tissue. Current techniques, such as voltage mapping, provide valuable insights; however, they are limited by the presence of numerous bystander areas and the occurrence of incomplete transmural scarring. Recent advancements in functional substrate mapping have focused on identifying critical isthmuses without requiring hemodynamic stabilization during VT, thereby shifting the emphasis to the analysis of potentials during baseline rhythm. While methods like isochronal late activation mapping have improved target identification, they primarily address conduction abnormalities without adequately considering repolarization heterogeneity. This review highlights emerging technologies that utilize unipolar potentials to assess repolarization heterogeneities and identify VT isthmuses. Furthermore, novel ablation sources such as pulsed-field ablation, bipolar ablation, and ultra-low temperature cryoablation are being explored to create deeper and more durable lesions, addressing the limitations of traditional radiofrequency ablation. These advancements aim to reduce VT recurrence and improve overall treatment efficacy. Ultimately, understanding these innovative strategies is expected to optimize procedural outcomes and significantly enhance the management of patients with scar-related VT.

## 1. Introduction

**Figure 1:**
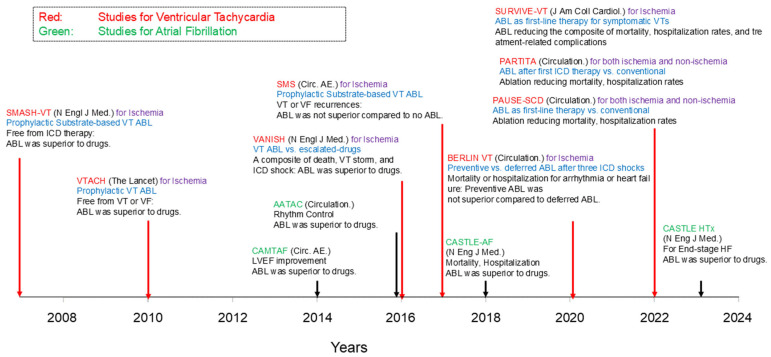
The history of large-scale clinical trials of catheter ablation for systolic heart failure. ABL indicates ablation; HF, heart failure; LVEF, left ventricular ejection fraction; VF; ventricular fibrillation; VT; ventricular tachycardia.

Catheter ablation for ventricular tachycardias (VTs), especially in scar-related VTs, in patients with heart failure and reduced ejection fraction is a key area of focus within non-pharmacological heart failure therapies. While several randomized controlled trials have demonstrated favorable prognostic outcomes for atrial fibrillation, evidence regarding the efficacy of catheter ablation in reducing cardiac mortality in patients with VTs remains inconclusive (Figure 1) [1,2,3,4,5,6,7,8]. Of course, differences in clinical conditions, as reflected by the PAINESD score (atrial fibrillation is often ablated during the compensated phase, whereas VT is frequently associated with hemodynamic instability during an electrical storm), influence prognosis [9]. Therefore, it is not appropriate to directly compare clinical outcomes following AF and VT ablation. In terms of procedural considerations, a major factor contributing to this uncertainty is the lack of well-established ablation strategies for VTs, including the optimal timing of ablation, procedural endpoints, and methods for visualizing ablation targets.

The principal challenges associated with VT ablation include (1) the difficulty in identifying suitable ablation targets and (2) the deep location of these targets within the myocardial tissue [10]. Concerning the first challenge, ablation strategies have been developed that focus on low-voltage areas, channels identified by delayed potentials, decremental evoked potentials, or local abnormal ventricular activities (LAVAs), defined as sharp, high-frequency ventricular potentials [11,12]. However, these techniques pose several challenges, including the requirement for prolonged procedural times to map these abnormal potentials throughout the entire ventricles and to achieve homogenization of the low-voltage areas [13]. Most importantly, if the VT presents with a focal pattern, which is more characteristic of non-ischemic cardiomyopathy rather than scar-related reentry, identifying the VT origin using these substrate mapping techniques becomes challenging [14]. In the context of scar-related VT, current three-dimensional mapping systems can identify areas of conduction delay during baseline rhythm, represented as zones of isochronal crowding, as demonstrated by isochronal late activation mapping (ILAM) [15]. However, these ablation methods targeting functional substrate abnormalities have still been demonstrating a recurrence rate of approximately 30% for VTs [16]. One of the major reasons for these residual VTs is the presence of critical isthmuses within the intramural layer. To address the second challenge, further investigation into needle ablation or pulsed-field ablation (PFA) is warranted [17,18].

This paper aims to review the current status of the technical challenges associated with catheter ablation for VTs and to highlight the latest promising technologies.

## 2. Current Status of Arrhythmogenic Substrate Mappings

Although activation mapping during tachycardia is the fundamental method for identifying reentrant circuits, most VTs cannot be sustained due to hemodynamic instability, particularly in patients with heart failure and reduced ejection fraction. While mechanical hemodynamic support, such as percutaneous left ventricular assist devices, can provide hemodynamic stability in these cases, evidence is lacking regarding the improvement of VT recurrence following the procedures [19,20]. Consequently, attention has shifted to functional substrate mapping during baseline rhythm, as these techniques can be performed without concern for worsening heart failure.

The currently accepted method for identifying arrhythmogenic substrates is voltage mapping, which utilizes bipolar potentials to indicate near-field scar areas and unipolar potentials to indicate far-field scar areas. Commonly accepted cut-off values for scar border zone are bipolar voltages of less than 1.5 mV and unipolar voltages of less than 8.3 mV in the left ventricle and less than 5.5 mV in the right ventricle [21,22]. The greatest advantage of these methods is their ease of acquisition and analysis. Dynamic voltage mapping has been reported as a useful tool for identifying the VT isthmus in regions where the voltage is recognized as <0.5 mV and classified as scar tissue. However, the widespread distribution of bystander areas remains a commonly acknowledged disadvantage. LAVAs or late potentials necessitate manual labeling with mapping tags; however, a recent study demonstrated that employing a frequency cut-off value of 220 Hz achieves high sensitivity and specificity for identifying late potentials or LAVAs [23,24]. Although pace mapping may assist in excluding bystander areas, technical issues related to pacing rates and coupling intervals still persist [25]. Scar area homogenization, achieved through scar dechanneling, may also serve as a potential resolution to bystander issues; however, the difficulty of completely eliminating all conducting channels has been reported, which can lead to VT recurrence [26].

Given these challenges, functionally guided substrate mapping has been the focus for improving the efficiency of VT mapping. Tung R. and colleagues reported the utility of ILAM in identifying critical isthmuses. Deceleration zones (DZs), defined as areas of the slowest conduction velocity, are characterized by the isochronal crowding of propagation within a scar, specifically where there is a clustering of isochrones (more than two isochrones within a 1 cm radius) [15]. Hattori M. and colleagues also demonstrated the significance of isochronal crowding within the VT isthmus, further reporting that the rotational activation pattern of propagation facilitates the identification of ablation targets [27]. These methods depend on bipolar potentials, which poses a limitation in identifying the region of interest if the VT isthmus is not located within the ventricular surface layers. Given the frequent presence of three-dimensional circuits in cardiomyopathies, it is necessary to develop functional substrate mapping techniques that utilize the relatively extensive information provided by unipolar potentials [28].

## 3. Prominent Mapping Techniques Using Unipolar Potentials

As described above, unipolar voltage mapping is well known for relatively reflecting extensive electrophysiological characteristics compared to bipolar mapping; however, functional analyses using unipolar potentials have not been previously assessed [29]. Furthermore, it is well established that two electrophysiological characteristics—conduction abnormalities and heterogeneity of refractoriness—are essential for the establishment of reentrant circuits [30]. However, previous functional substrate mappings, such as ILAM and the rotational activation pattern, have focused on conduction abnormalities but have not adequately addressed repolarization abnormalities. To address these issues, two similar yet distinct techniques utilizing unipolar leads have been reported in recent years.

The reentry vulnerability index reported by Orini M. and colleagues is proposed as a method for identifying VT circuits, reflecting activation time, activation–recovery interval (ARI), and repolarization time using unipolar potentials [31]. They reported that the index accurately localized 72% of VT origins, which is comparable to the DZs identified by ILAM [15,31]. Furthermore, Trayanova N. and colleagues reported efficient ablation outcomes characterized by fewer ablation sites and smaller ablation areas compared to conventional techniques [32]. However, this method has a significant drawback in that it does not clearly define the high-pass filter settings when using unipolar potentials. Commercially available mapping systems typically use a nominal high-pass filter setting of 2 Hz to cancel baseline drift. However, setting the filter at 2 Hz has been reported to critically impact the measurement of the ARI, which is essential for evaluating the ST-T segment [33]. In fact, we also observed distinct ARI measurements between the high-pass filter set at 2 Hz and at 0.05 Hz, which is consistent with the settings used for surface electrocardiograms [34].

**Figure 2:**
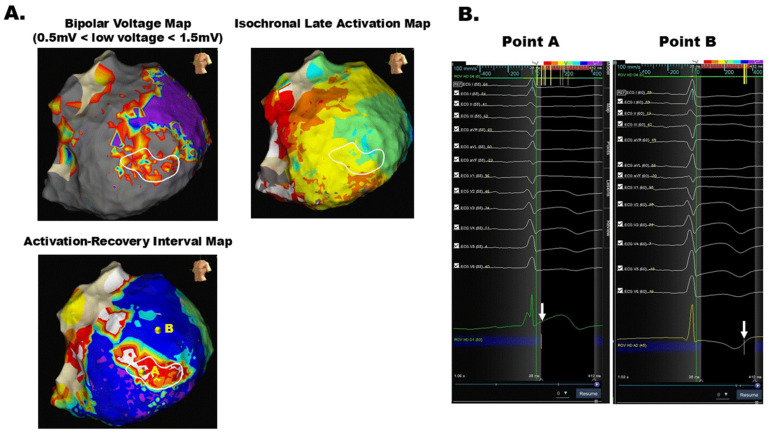
(**A**) Comparison of the bipolar voltage map, isochronal late activation map (ILAM), and activation–recovery interval (ARI) map of the epicardium in a case of ventricular tachycardia (VT). White circles indicate areas where diastolic potentials were recorded during VT. While both the voltage map and ILAM did not accurately identify regions with recorded diastolic potentials, the ARI map effectively identified the area. (**B**) Unipolar potentials of the epicardium from regions with shorter and longer ARI are shown. Local ARI can be automatically measured by aligning the starting point with the S-wave in lead I and defining the endpoint as the point of maximal dV/dt of the unipolar potentials, with a blanking period set to extend until the termination of the QRS complex. Point A represents the shortest ARI on the ARI map, while Point B represents the longest ARI. White arrows indicate the automatically documented endpoints.

It is well established that the refractory period of the ventricular myocardium correlates closely with the ARI measured using the Wyatt method on electrocardiograms recorded with the high-pass filter setting of 0.05 Hz [35]. Nagase S. and colleagues have consistently reported the utility of assessing unipolar potentials recorded with a high-pass filter setting of 0.05 Hz while setting the indifferent electrode to the inferior vena cava [34]. Localized J-ST elevation with a negative T-wave in unipolar potentials, such as the coved type J-ST segment in surface electrocardiograms, can be identified on the epicardium in cases of Brugada syndrome [36]. They concluded that the area exhibiting localized coved type J-ST segments indicates arrhythmogenic substrates associated with Brugada syndrome or J-wave syndrome [37]. Moreover, we reported the utility of unipolar potentials in identifying the trigger area of ventricular fibrillation in a case of Brugada syndrome [38]. Furthermore, recent analyses have revealed that delayed potentials in bipolar potentials, typically interpreted as conduction abnormalities, may actually have different underlying mechanisms in cardiomyopathies and Brugada syndrome [39]. Based on these findings, we have proposed a novel method for visualizing ARI heterogeneities in the ventricles [34]. This method automatically calculates the ARI from the start, defined as the end of the QRS complex, to the end, defined as the point of maximal dV/dt of the unipolar potentials automatically, which is consistent with the Wyatt method (Figure 2).

This novel mapping technique, using the Advisor HD Grid Mapping Catheter (Abbott, MN, USA) with the EnSite system, can be automatically constructed by utilizing unipolar potential morphologies obtained during sinus rhythm or consistent ventricular pacing. Our feasibility study demonstrated that regions with a short refractory period identified using this method exhibited a comparable probability of identifying VT origins to the DZs identified by ILAM. Notably, this approach successfully identified VT origins in approximately 70% of cases where ILAM failed to do so. The findings may be influenced by differences in electrode types: ILAM, derived from bipolar potentials, primarily reflects near-field information and thus has limitations in detecting three-dimensional VT circuits with intramural isthmuses. In contrast, ARI mapping, based on unipolar potentials, may capture far-field information, offering an advantage in cases with intramural VT isthmuses. Based on this speculation, the ARI mapping technique may be effective for cases in which the VT origin cannot be identified using conventional techniques focused on conduction delay. Although several imaging techniques, including computed tomography and magnetic resonance imaging, can provide supportive information for identifying the ventricular tachycardia isthmus prior to the procedure, direct contact mapping remains a crucial method for identifying arrhythmogenic substrates [40]. Imaging quality, particularly in patients with cardiac implantable electronic devices such as implantable cardioverter defibrillators, will be a critical limitation in accurately identifying the details of VT origins [41]. These new technologies focusing on repolarization abnormalities will enhance our understanding of the reentrant circuits of VTs, complementing the widely accepted methods that concentrate on conduction delays, such as the DZs identified by ILAM.

## 4. Current Status and Future Perspectives of Ablation Sources

Although some of the above-described mapping techniques have been adapted for procedures, VT recurrences remain significant in both ischemic and non-ischemic patients. The primary reason for this is the complexity of the three-dimensional circuit, which includes subendocardial, subepicardial, intramural, and transmural components. In general, radiofrequency ablation creates scar tissue within 4 mm of the electrode, leading to incomplete transmural scarring [42].

For addressing this issue, new treatment approaches such as bipolar ablation, ultra-low temperature cryoablation, needle-tipped electrode ablation, radiation therapy, or PFA have been proposed. Bipolar ablation utilizes two ablation catheters connected to a radiofrequency generator, with one catheter attached to the output terminal and the other to the ground reference. The two catheters are positioned on opposite surfaces of the myocardial layers, particularly across the ventricular septum [43]. The ultra-low temperature cryoablation system utilizes a 15 mm cryoablation element and is considered well suited for papillary muscle and moderator band-related VTs, as it adheres to the tissue and can create deeper and larger lesions compared to conventional cryoablation systems [44,45]. Needle ablation has evidence in humans showing that it typically creates lesions ranging from 5 to 20 mm in size, with a low incidence of complications and a procedural success rate of 97% [17]. Stereotactic ablative radiotherapy is recognized as a noninvasive method for VT ablation using ionizing radiation, which induces DNA breaks in the tissues leading to cell death. Target zones are created within a three-dimensional coordinate system based on computed tomography [46].

Among these cutting-edge innovations, PFA is anticipated to be one of the most versatile techniques available. The concept of PFA involves creating irreversible pores in the cell membrane through the application of a direct external current, resulting in the selective death of cardiac myocytes [47]. This technique has already been widely adopted for atrial ablation in various countries across Europe and North America; however, attempts at VT ablation have only just begun [48,49]. PFA enables extensive ablation in a short duration; however, it has been noted that the depth of lesions may be shallower compared to conventional radiofrequency ablation, which could represent a significant disadvantage, particularly in the context of VT [49]. Conversely, experimental studies have demonstrated lesion depths in the ventricles ranging from 4 to 8 mm with PFA, suggesting that this technique may be comparable to, or even deeper than, radiofrequency ablation [50]. Previous case series have demonstrated a procedural success rate of over 80% with no recurrence of VTs [51]. Notably, contact force, which was once deemed to be of limited importance in the early days of PFA, is now recognized as essential for creating deep and large lesions with this technique [52]. The uniquely shaped 9 mm focal spherical lattice tip catheter, AFFERA (Medtronic, Minneapolis, MN), may enhance the contact force of the tip compared to that of other ringed or flower-shaped catheters. A critical consideration when adapting PFA for the outflow tract area is the reported occurrence of transient ST-segment depressions in surface electrocardiograms [53]. Therefore, coronary evaluations may be necessary prior to procedures that target the outflow tract.

**Figure 3:**
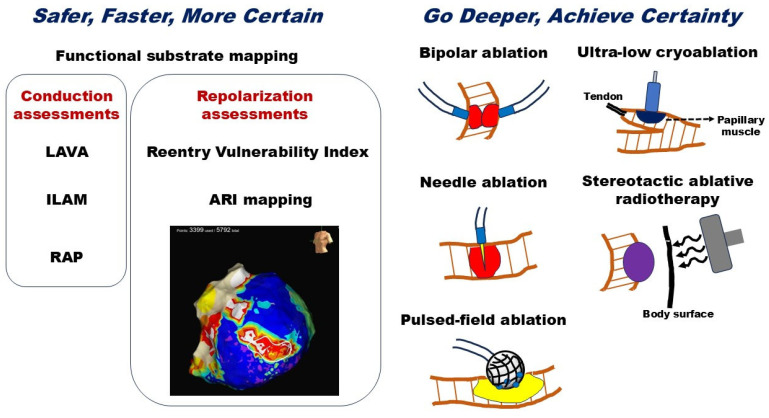
Current status and future perspectives of ventricular tachycardia ablation. The ARI map represents epicardial surface mapping in a case of VT. The encircled area on the ARI map highlights the critical isthmus of the VT circuit. ARI indicates the activation–recovery interval; ILAM, isochronal late activation map; LAVA, local abnormal ventricular activities; RAP, rotational activation pattern. The right figure indicates complications associated with ablation procedures.

These novel sources for ablation may aid in suppressing VT recurrence and contribute to reducing the necessity for implantable cardioverter defibrillator therapy and cardiac mortality (Figure 3).

Several complications following VT ablation continue to be a concern in the current era. A recent study reported that the procedure-related mortality rate within 30 days following VT ablation in patients with reduced ejection fraction was 0.4%, with complications including stroke due to thromboembolism, cardiac tamponade, pericardial effusion, hematoma, and pericarditis, the most frequent of which was hematoma [54]. To prevent hemorrhage at the puncture sites, vascular closure devices can serve as effective tools when used in conjunction with an echo-guided puncture technique [55]. The safety of PFA in cases of atrial fibrillation has been established; thus, there are strong expectations for enhanced safety in its application for VT ablation as well [56].

## 5. Conclusions

The procedural and prognostic outcomes of VT ablation remain suboptimal. In recent years, functional substrate mapping has advanced to assess not only conduction abnormalities but also repolarization heterogeneity, ushering in a new era in identifying arrhythmic substrates. To achieve deeper and more durable lesions, various ablation technologies have been developed. The innovations discussed in this paper are expected to significantly enhance treatment efficacy.

## Abbreviations

ARI	activation–recovery interval
DZ	deceleration zone
ILAM	isochronal late activation map
LAVA	local abnormal ventricular activities
PFA	pulsed-field ablation
VT	ventricular tachycardia
